# Axonal Noise as a Source of Synaptic Variability

**DOI:** 10.1371/journal.pcbi.1003615

**Published:** 2014-05-08

**Authors:** Ali Neishabouri, A. Aldo Faisal

**Affiliations:** 1 Department of Bioengineering, Imperial College London, London, United Kingdom; 2 Department of Computing, Imperial College London, London, United Kingdom; 3 MRC Clinical Sciences Centre, London, United Kingdom; University College London, United Kingdom

## Abstract

Post-synaptic potential (PSP) variability is typically attributed to mechanisms inside synapses, yet recent advances in experimental methods and biophysical understanding have led us to reconsider the role of axons as highly reliable transmission channels. We show that in many thin axons of our brain, the action potential (AP) waveform and thus the Ca^++^ signal controlling vesicle release at synapses will be significantly affected by the inherent variability of ion channel gating. We investigate how and to what extent fluctuations in the AP waveform explain observed PSP variability. Using both biophysical theory and stochastic simulations of central and peripheral nervous system axons from vertebrates and invertebrates, we show that channel noise in thin axons (<1 µm diameter) causes random fluctuations in AP waveforms. AP height and width, both experimentally characterised parameters of post-synaptic response amplitude, vary e.g. by up to 20 mV and 0.5 ms while a single AP propagates in C-fibre axons. We show how AP height and width variabilities increase with a ¾ power-law as diameter decreases and translate these fluctuations into post-synaptic response variability using biophysical data and models of synaptic transmission. We find for example that for mammalian unmyelinated axons with 0.2 µm diameter (matching cerebellar parallel fibres) axonal noise alone can explain half of the PSP variability in cerebellar synapses. We conclude that axonal variability may have considerable impact on synaptic response variability. Thus, in many experimental frameworks investigating synaptic transmission through paired-cell recordings or extracellular stimulation of presynaptic neurons, causes of variability may have been confounded. We thereby show how bottom-up aggregation of molecular noise sources contributes to our understanding of variability observed at higher levels of biological organisation.

## Author Summary

The fundamental signal of the nervous system is the action potential: an electrical spike propagated along neurons and transmitted between them via synapses. Once triggered, action potentials are generally assumed to be robust to noise, and the variability observed at all levels of the nervous system is primarily attributed to synapses. However, this view is based on data from classically studied axons, which are very large compared to the average diameter of axons in the mammalian nervous system, and even larger when compared to the thinnest axons. As the effects of thermodynamic noise affecting the proteins responsible for the initiation and propagation of action potentials are much bigger in thin axons, the assumption does not necessarily hold for very thin axons. We show that the action potentials waveform in thin axons is subject to random variability. Fluctuations in this waveform result in fluctuations in synaptic ionic currents, and account for a significant portion of the variability observed at the synapse.

## Introduction

The great majority of axons use action potentials (APs) to transmit information reliably to synapses. Once the AP arrives at the synapse the characteristics of its waveform are fundamental in determining the strength and reliability of information transmission, as was extensively shown in the central and peripheral nervous system of both vertebrates and invertebrates [1]–[14]. Although the nervous system exhibits stochastic variability (noise) at all levels (see [15] for a review), it is generally assumed that little random variability affects the AP waveform as it travels from the soma along the axon to the synapse. However, recent understanding of biophysics and experimental methods prompt us to reconsider this common assumption.

The AP is mediated by voltage-gated ion channels, which control the flow of ionic currents through the membrane. Thermodynamic fluctuations in voltage-gated ion channels result in probabilistic gating, producing random electrical currents called channel noise [16]. In thin axons, the behaviour of individual ion channels can have significant effects on the membrane potential dynamics due to the higher input resistance of those axons [17]–[19]. Fewer channels sustain AP conduction and fluctuations in individual ion channels have a larger impact on the membrane potential in thinner axons. Faisal et al. [20] have shown that channel noise sets a lower limit to reliable axonal communication at 0.08–0.1 µm diameter, a general limit matched by anatomical data across species. Above this limit, in axons of 0.1–0.5 µm diameter, channel noise causes variability in the rising phase of the AP and the resting input resistance of axons. Therefore APs are jittered, shifted, added and deleted in a history-dependent way along the axon [18]. Thus, noise in axons affects the timing of APs and therefore reduces the information capacity of the neural code. Here, we are going to investigate how noise in axons affects the waveform of APs, and produces random variability in the responses of synapses, with implications for information transmission and learning.

Attempts at investigating the impact of axonal noise on the synapse have so far been limited to rather large diameter axons (≥1 µm diameter) [21], [22]. However, many unmyelinated axons are very thin (0.1–0.3 µm diameter [23]). Examples include cerebellar parallel fibres (average diameter 0.2 µm [24]), C-fibres implicated in sensory and pain transmission (diameter range 0.1–0.2 µm [25]) and cortical pyramidal cell axon collaterals (average diameter 0.3 µm [26], making up most of the local cortical connectivity [26]). The variability of the AP waveform in all these axons is unknown. Basic biophysical considerations suggest that axonal noise sources are bound to introduce fluctuations [20], [27] in the shape of the travelling AP waveform in thin axons with immediate consequences for synaptic transmission and reliability [28].

Intracellular recordings from such thin axons are difficult to obtain. Extracellular stimulation offers only limited signal resolution and stimulus control, and tiny intracellular volumes limit the application of imaging methods to quantify AP waveforms accurately. This motivated the study presented here which uses biophysically detailed stochastic simulations of travelling APs in thin axons and basic biophysical theory. Our goal is to investigate the mechanisms behind the observed synaptic variability; specifically how much variability can be explained by channel noise in axons. We quantify waveform fluctuations of single propagating APs in terms of standard synaptic efficacy measures, namely AP width and height. We explain how channel noise causes AP waveform fluctuations and show that these fluctuations scale with axon diameter according to an inverse power law, i.e. the finer an axon the bigger the impact. We then investigate the AP waveform fluctuations for propagating spike trains and predict the post-synaptic response variability axonal noise would cause in two ways: 1. by using models of synaptic dynamics and vesicle release and 2. by using direct experimental data linking AP waveform to post-synaptic response. Thus, we will be able to estimate the influence of axonal channel noise on synaptic variability.

## Results

### Action potential waveform variability

**Figure 1:**
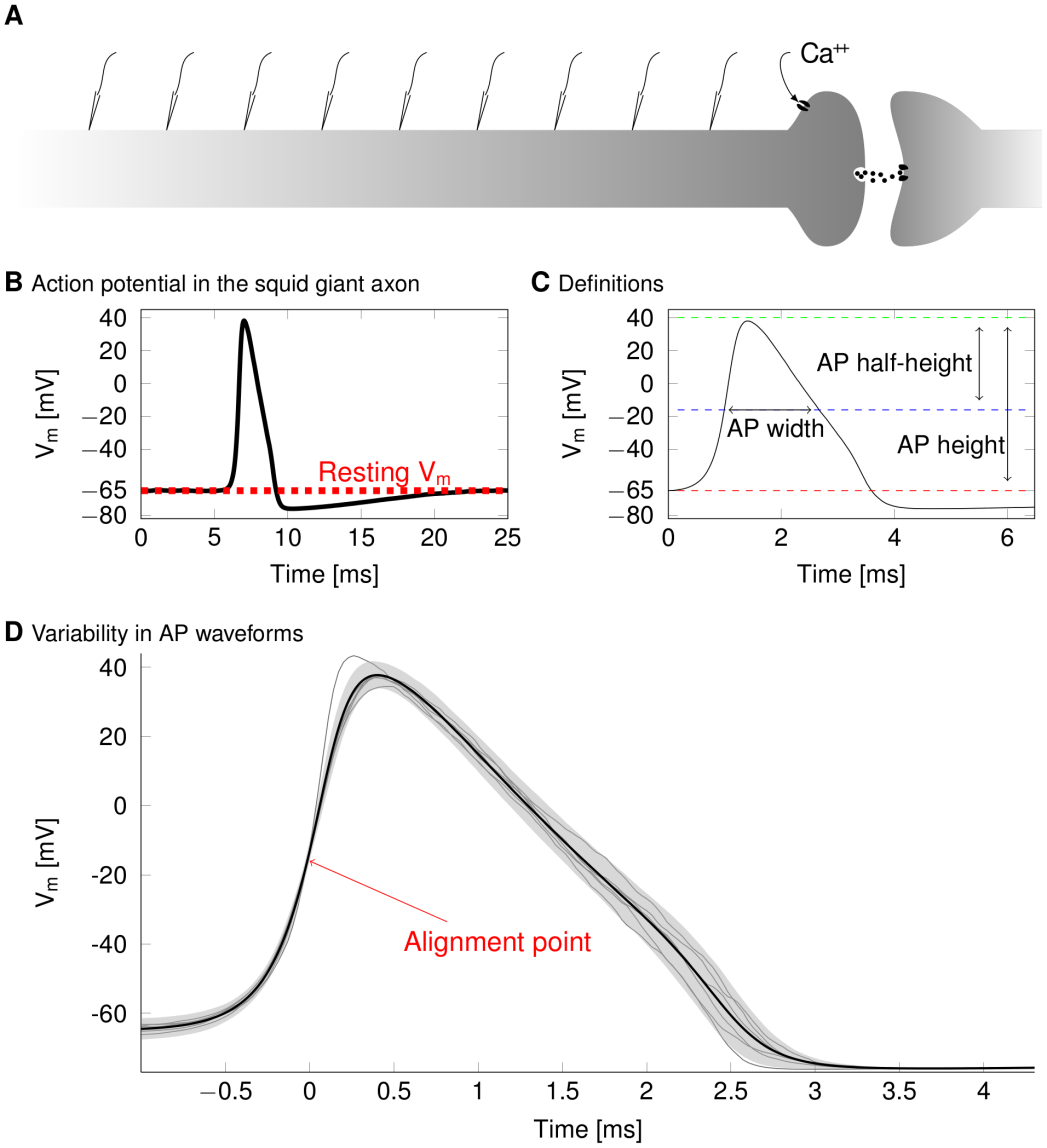
Definitions and methods. (A) We record membrane potential and ionic currents at regularly placed points along the axon, and compare the (B) shape of the AP waveforms (black) and the resting membrane potential (dashed red). (C) Action potential features are determined individually for each AP. The amplitude of the AP is defined as the maximum membrane potential. The width is the delay between the crossings of the mid-height level. (D) AP shape fluctuations. Due to channel noise, APs triggered in an identical fashion will have different shapes across trials. Here APs in a 0.2 µm diameter axon from 5 trials out of 250 are superimposed. The point at which the membrane potential crossed the half-height line is used to align APs.

We find that single APs propagating in central and peripheral nervous systems (CNS and PNS), mammalian and invertebrate axons of up to 1 µm diameter display large random variability in their waveform as they propagate. We visualize this by measuring the AP waveform (membrane potential versus time) at various positions along the axon (Figure 1.A,B) and then align the waveforms at the instant of half-peak crossing (Figure 1.D). As a control, we simulated a deterministic axon, i.e. one that had the same set of biophysical parameters and received the same stimuli but where we modelled the ion channels using deterministic kinetics instead of the corresponding stochastic kinetics [19], [29]. APs in all our deterministic simulations, starting from the same initial condition and receiving the same trigger input, exhibit no waveform variability across repeated trials. Since the stochastic kinetics of ion channels are the only source of variability given that all other parameters and stimuli are controlled by our simulation, the variability of the travelling AP waveform observed must be due to channel noise and thus entirely random in nature. Crucially, the AP waveform is not only variable across repeated trials with identical stimulus, but also varies as the same AP propagates along the axon.

**Figure 2:**
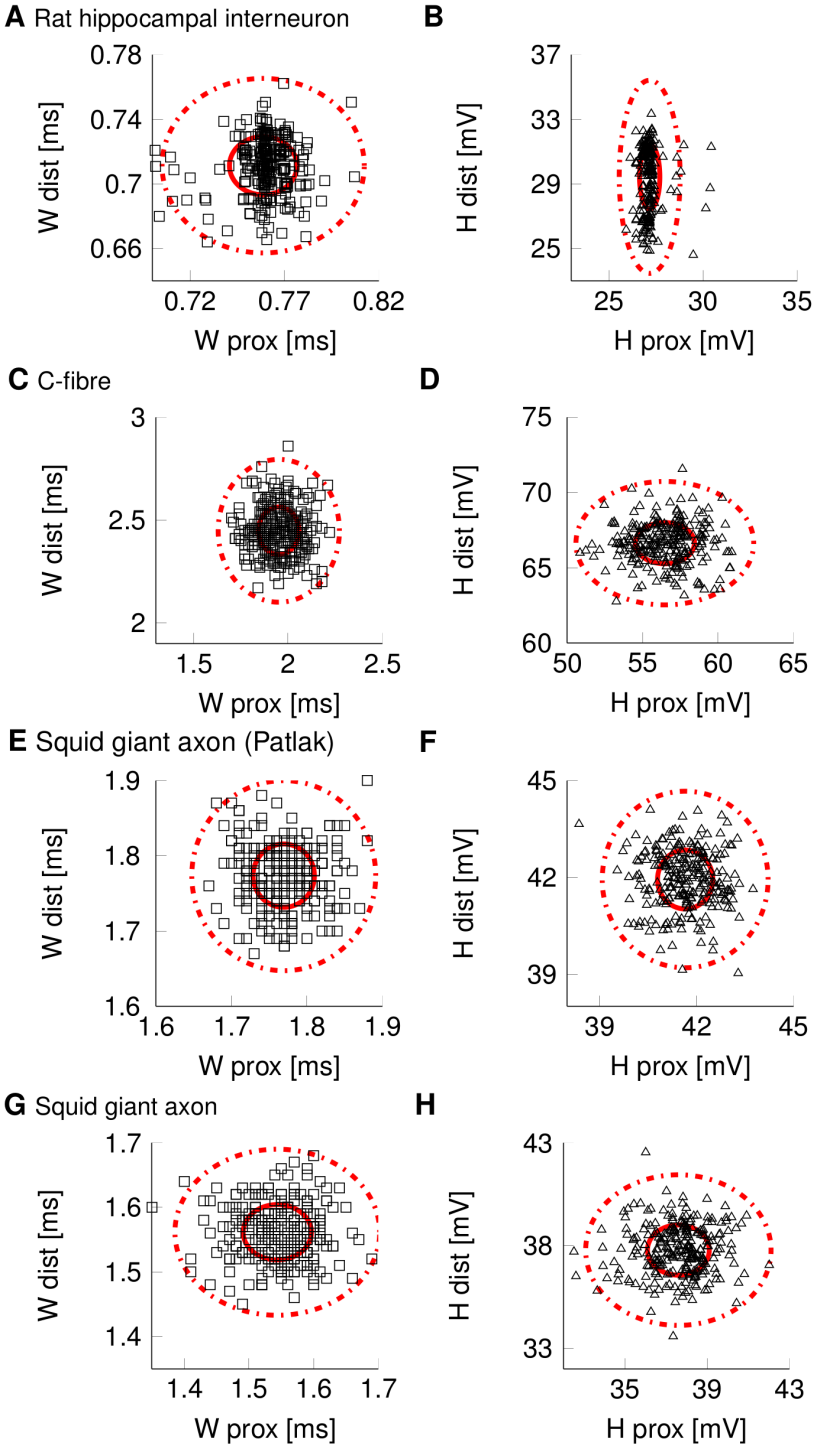
Random variability of AP waveform in thin axons (d = 0.2 µm) of four different types. Subfigures display data for N>200 single APs triggered by identical stimuli and initial conditions (thick circle, 1×SD; dotted circle 3×SD). (A) Distribution of AP width and (B) AP height for rat hippocampal interneuron model axon. (C) Distribution of AP width and (D) AP height for C-fibre axons. (E) Distribution of AP width and (F) AP height for squid giant axons with Patlak channels. (G) Distribution of AP width and (H) AP height in squid giant axons.

**Figure 3:**
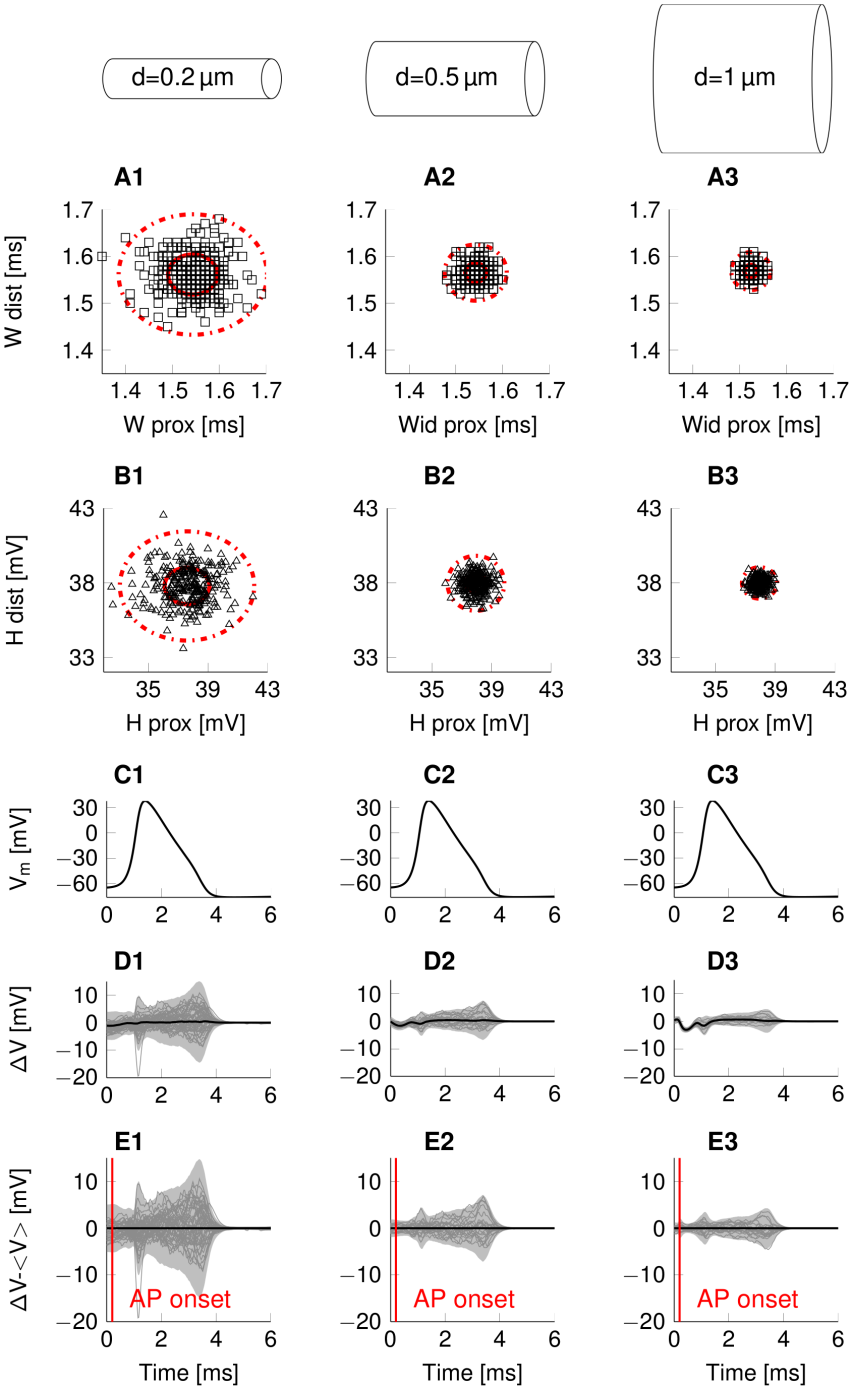
Random variability of AP waveform in thin squid giant axon type axons of 3 diameters. The subfigures display data for N>200 single APs triggered by identical stimuli and initial conditions in thin squid giant axon model. (A) Distribution of AP width and (B) AP height (red circle, 1×SD; dotted circle 3×SD). (C) Mean waveform of the AP at the proximal site. (D) Pairwise difference between an AP's shape at the proximal and the distal location. The average difference is plotted in thick black, while the light grey shaded area represents the 3×SD range. Grey lines represent sample traces plotted individually. (E) Fluctuations around the mean pairwise difference. The average difference is plotted in thick black (0 by definition), while the light grey shaded area represents the 3×SD range. Grey lines represent sample traces plotted individually.

Comparing the variability of the AP waveform in axons with identical biophysical parameters and ion channels but varying axon diameter from 0.1 µm to 1 µm, shows that the waveform fluctuations become larger as the axon becomes thinner. This is true for both models of squid giant axons, rat hippocampal interneuron and C-fibre axons. The general structure of the variability profile remains preserved across diameters. The width of the propagating AP varies as it travels down a thin axon in the order of a tenth of a millisecond (Figure 2.A,C,E,G). Similarly, AP height varies in the order of 1 to 10 millivolts (Figure 2.B,D,F,H). The variability is more pronounced the thinner the axon is (Figure 3.A,B). The variations between proximal and distal AP shape are, as expected uncorrelated (R^2^<<0.2 across all diameters and axons for both AP heights and AP widths). This implies that both AP width and height become decorrelated with themselves (autocorrelation decreases) and between each other (cross-correlation decreases) the further the AP propagates down an axon (Supplementary Figure S1).

#### Deterministic transformation of the propagating AP waveform

The AP waveform changes in a deterministic way after AP initiation [30] (Figure 3.C,D). The AP upstroke becomes steeper as the AP travels away from the AP initiation site and approaches its steady-state shape after several length constants (after about 1 mm in a 0.2 µm diameter axon). The change in the average AP waveform of the stochastic simulation matches the AP waveform change of the corresponding deterministic simulated axon [31]. This is because close to the proximal stimulation site, the AP shape is determined by the stimulus driving a fully resting axon, and the response speed is limited by the resting membrane's time constant. However, once the AP propagates the depolarisation is driven by the APs own axial current, which acts over the broad region (about one length constant 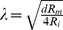, which is a property of the passive axonal membrane and represents how well a subthreshold potential spreads along the axon) of the rising phase of the AP (see also [18]). Na^+^ conductance increases across this region due to the opening of voltage-gated channels in response to the rising phase of the AP. Therefore, the rise time of the travelling AP is faster than the rise time of the AP at the stimulus initiation site. The effect is more pronounced the thicker the axon is, because the larger cross-sectional area lowers the axial resistance in proportion to the axonal cross-section area (which is quadratic in the diameter). The deterministic transformation of the AP waveform is predictable, reproducible and needs not cause loss of information about the generating stimulus.

#### Stochastic variation of the propagating AP waveform

Axonal channel noise introduces non-deterministic, random variability in the propagating AP waveform. This stochastic component of waveform change has similar size as the deterministic component at the largest diameter but unlike the deterministic effect, its amplitude increases as diameter decreases (Figure 3.E). The stochastic effect is non-predictable and cannot be compensated for at the synapses. We find AP waveform fluctuations, quantified here in standard deviations around the mean waveform are up to 3 times larger in amplitude than resting membrane potential fluctuations caused by channel noise (Figure 3.E, between the highest variability point at approx. 3 ms and the variability at t = 0, before the onset of the AP). Thus, in thin axons, the AP mechanism itself enhances waveform variability due to channel noise.

**Figure 4:**
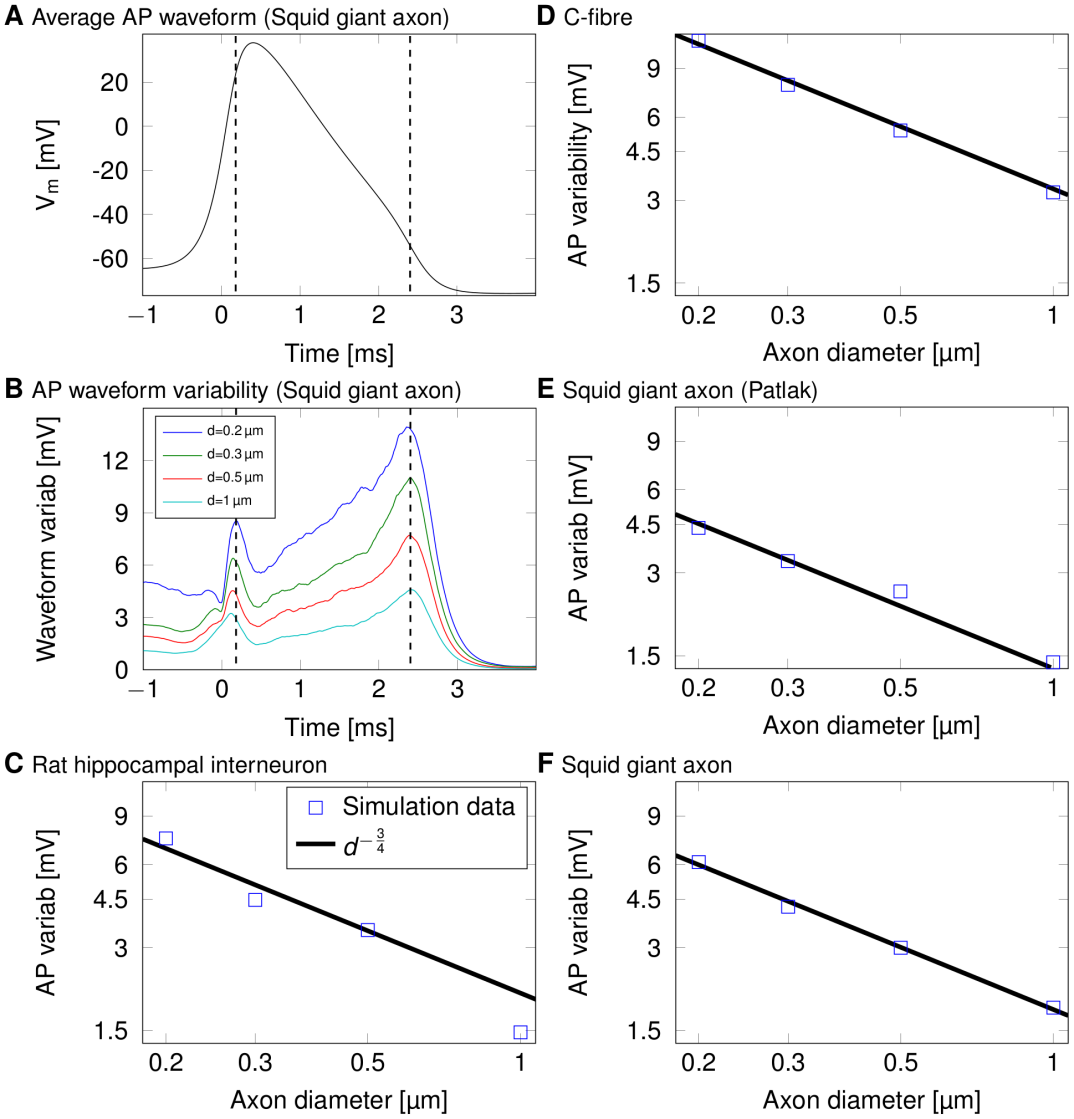
Travelling APs' waveform fluctuations scale with axonal diameter with an inverse power low. (A) Typical shape of an action potential in the squid giant axon. (B) The variability in the waveform at each moment in time (N = 250). We define the variability as 3×SD of the membrane potential at each point in time. (C) Log-log plot of 3×SD of fluctuations in AP shape over diameter for the rat hippocampal interneuron. (D) Log-log plot of 3×SD of fluctuations in AP shape over diameter for a C-fibre axon. (E) Log-log plot of 3×SD of fluctuations in AP shape over diameter for a squid giant axon (Patlak channels). (F) Log-log plot of 3×SD of fluctuations in AP shape over diameter for a squid giant axon.

**Figure 5:**
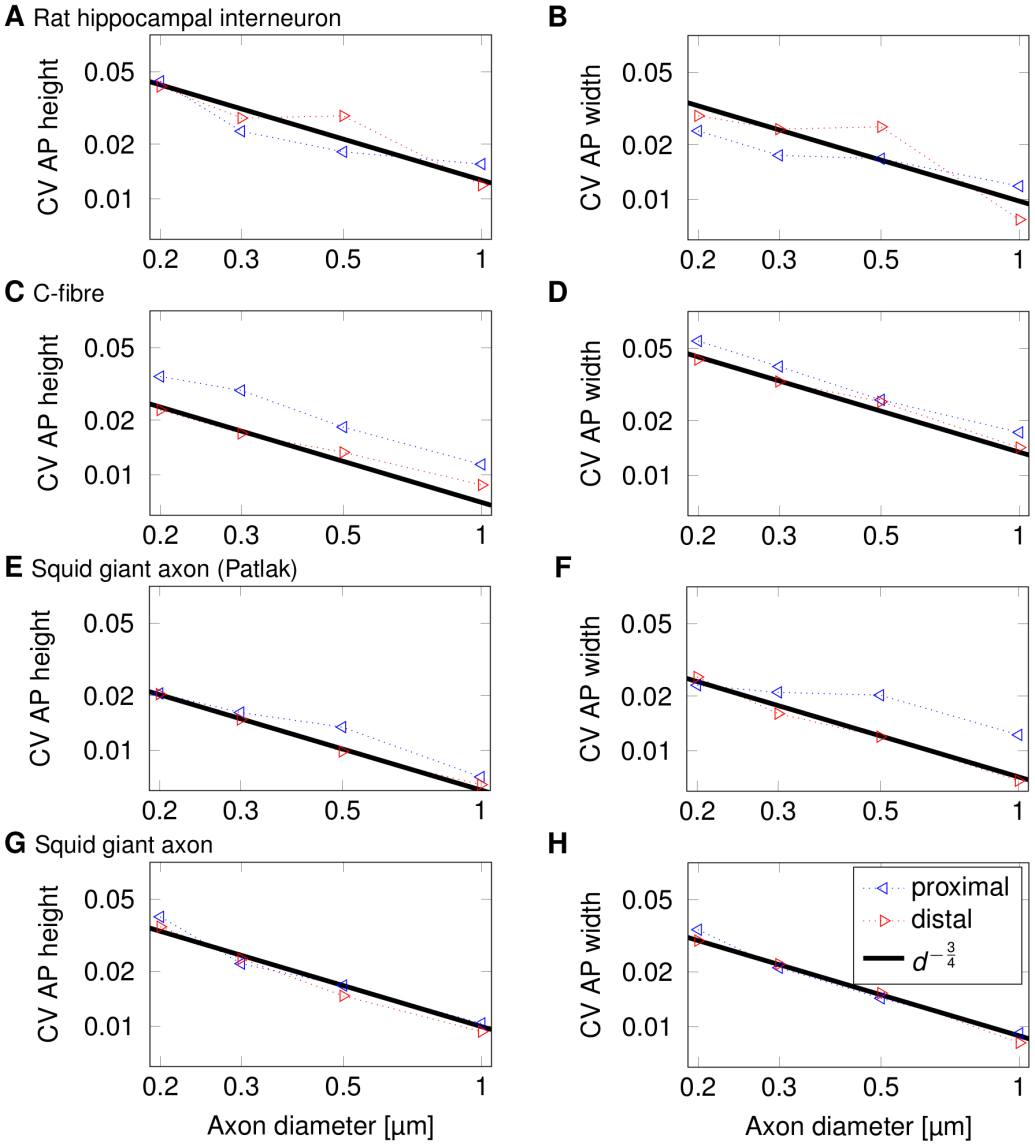
Variability of the AP width and height scales with axon diameter. The power-law relationship is valid at both proximal (left-pointed triangle) and distal (right-pointed triangle) points in all four types of axons. (A) Log-log plot of CV of AP height over diameter for a rat hippocampal interneuron. (B) Log-log plot of CV of AP width over diameter for a rat hippocampal interneuron. (C) Log-log plot of CV of AP height over diameter for a c-fibre axon. (D) Log-log plot of CV of AP width over diameter for a c-fibre axon. (E) Log-log plot of CV of AP height over diameter for a squid giant axon with Patlak channels. (F) Log-log plot of CV of AP width over diameter for a squid giant axon with Patlak channels. (G) Log-log plot of CV of AP height over diameter for a squid giant axon. (H) Log-log plot of CV of AP width over diameter for a squid giant axon.

This random variability of the AP waveform does not only result from AP initiation variability, but is also actively generated as the AP propagates along thin axons. The amplitude of waveform fluctuations (as defined by the standard deviation of the membrane potential at each point in time, see Figure 4.B) increases over-proportionally as axon diameter decreases below 1 µm (Figure 4.C–F). Plotting the relative variability as the coefficient of variation (CV, defined as the ratio of the standard deviation over the mean) of AP width or height, over axon diameter reveals a common power-law relationship of 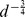 [18]. The power law holds for the height of APs and their widths (Figure 5), as well as the fluctuations in the overall shape of APs (Figure 4). These relationships can be understood by analysing how the AP mechanism and the ion channel's stochastic nature produce the observed AP waveform variability and have been derived by Faisal et al. [18]. As they show, the ratio of membrane fluctuations over total number of channels is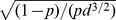, where 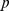 is the open channel probability. Therefore the relative effects of channel fluctuations grows as 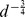.

### Underlying mechanisms of waveform variability

**Figure 6:**
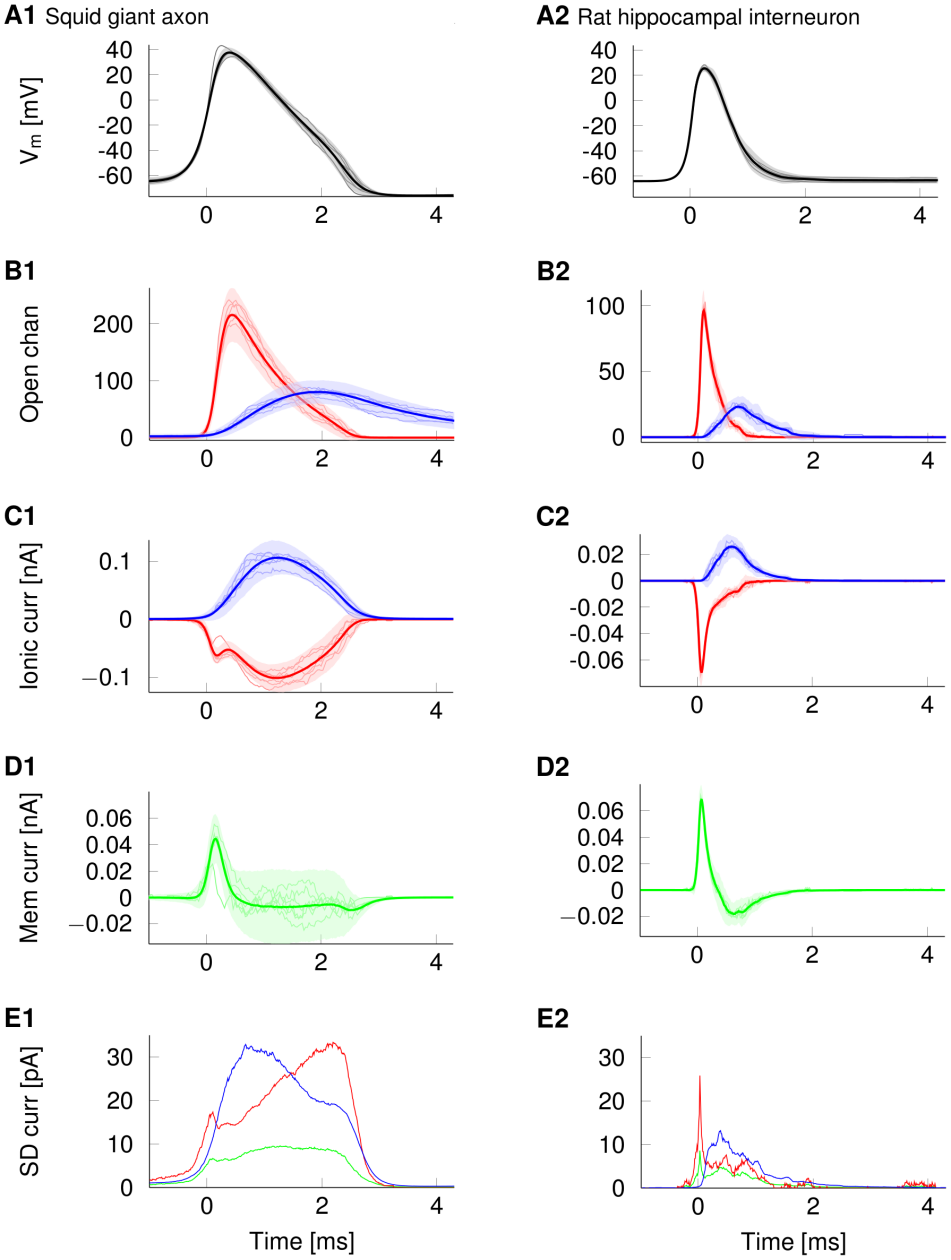
Ion channel and current fluctuations underlie waveform variability. Ion channel and current fluctuations in an AP travelling along 0.2 µm diameter axons. All traces are aligned at the instant when the rising AP crosses half-peak. Data from the squid giant axon model is on the left, while the right plots show data from the rat hippocampal interneuron model. Shaded areas in A–D are the 3×SD envelope around the mean curve (dark curve). Light curves in A–D represent a sample of individual traces. (A) Membrane potential waveforms (B) Number of open Na^+^ (red) and K^+^ (blue) channels (C) Current flowing through Na+ (red) and K^+^ (blue) channels (D) Net membrane current (sum of Na^+^, K^+^ and leak) (E) SD of Na^+^ (red), K^+^ (blue) and net membrane (green) currents.

To measure how channel noise affects the propagating waveform, one has to track the relevant quantities at corresponding points of the moving AP. To this end, the time series recorded at closely spaced axonal positions (here, corresponding to a cylindrical membrane compartment of the axon model) are superimposed after having been aligned at the instant when the membrane potential crosses its half AP peak value. Thus, the individual quantities and their variability at corresponding points of the travelling AP are displayed at corresponding points (Figure 6).

The variability of the waveform has a characteristic structure that is conserved across different axons types and diameters as it is caused by the basic mechanism of the AP itself. The first maximum in waveform variability is reached in the late rising phase of the AP (between half peak and peak depolarisation, see Figure 4.A,B). The location of this peak is not an artefact of our aligning of APs (at 50% AP height, c.f. alignment at 20% AP height in Supplementary Figure S2). This first peak is due to fluctuations in the number of opening Na^+^ channels and Na^+^ current (red curves in Figure 6.B,C), as the first peak of Na^+^ current variability is reached at half-peak membrane depolarisation (Figure 6.C, shortly after 0 ms). The variability of the depolarising Na^+^ current accounts for the variations in AP height because the number of Na^+^ open channels and their inactivation prior to reaching Na^+^ reversal potential (the upper limit to AP peak) determine how much driving current is depolarising the membrane capacitance. K^+^ channels begin to open later, and thus Na^+^ channels carry most of the net membrane current in this initial phase of the AP and are responsible for the initial variability (Standard deviation (SD) profile of Na^+^ current, red curve, and K^+^ current, blue curve, with net membrane current, green curve, in Figure 6.E).

The second, broad peak in waveform variability is reached in the repolarizing phase (Figure 6.A and Figure 4.A,B beginning at 1 ms and increasing up to 2.5 ms). As the rate of repolarization (here, <50 mV/ms) is much slower than that of depolarization (here, >200 mV/ms), variability in the height of the AP waveform translates into much larger changes of AP width. Note, AP width is measured between the up and down crossings of any given membrane potential level, here chosen to be half-peak depolarization. Thus, AP width variability is mainly generated in the repolarizing phase of the AP and caused by a long period of large fluctuations in net membrane current (Figure 6.D, between 0.75 and 2.25 ms). Variability is generated initially by K^+^ current noise and then by Na^+^ current noise (Na^+^, red, and K^+^, blue, in Figure 6.B,C). After K^+^ channels begin to open in the early repolarizing phase, K^+^ current fluctuations peak as K^+^ channel opening probabilities increase and the variance of the number of open channels becomes larger. The increase in variance can be understood, if one considers that a population of 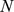 ion channels with open probability 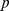 follows a binomial distribution for the number of open channels. The variance in the number of open channels is given by 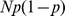 and thus has a maximum as the open probability 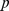 approaches 0.5 from all channels closed (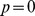) or all channels open (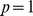). By the time the maximum K^+^ channel open probability is reached (which is not necessarily 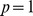 for many voltage-gated ion channels), electro-motive forces are lower than near AP peak and, membrane potential fluctuations due to K^+^ currents have consequently lower amplitudes. An equally large and broad maximum in the fluctuations is due to Na^+^ channel inactivation in the late repolarizing phase for analogous reasons, following a similar binomial argument, when Na^+^ electro-motive forces are large.

Thus, AP height variability (Figure 5.A,C,E,G) is mainly caused by the fluctuating number of open and inactivating Na^+^ channels during the upstroke of the AP. AP width variability (Figure 5.B,D,F,H) is predominantly caused by the noisy repolarizing phase of the AP, where both K^+^ and Na^+^ channels contribute to large fluctuations in the rate of repolarization. Having described how channel noise affects a single AP's waveform, the question arises whether AP waveforms are more variable in spike trains, as APs may influence each other.

### Waveform variability in spike trains

**Figure 7:**
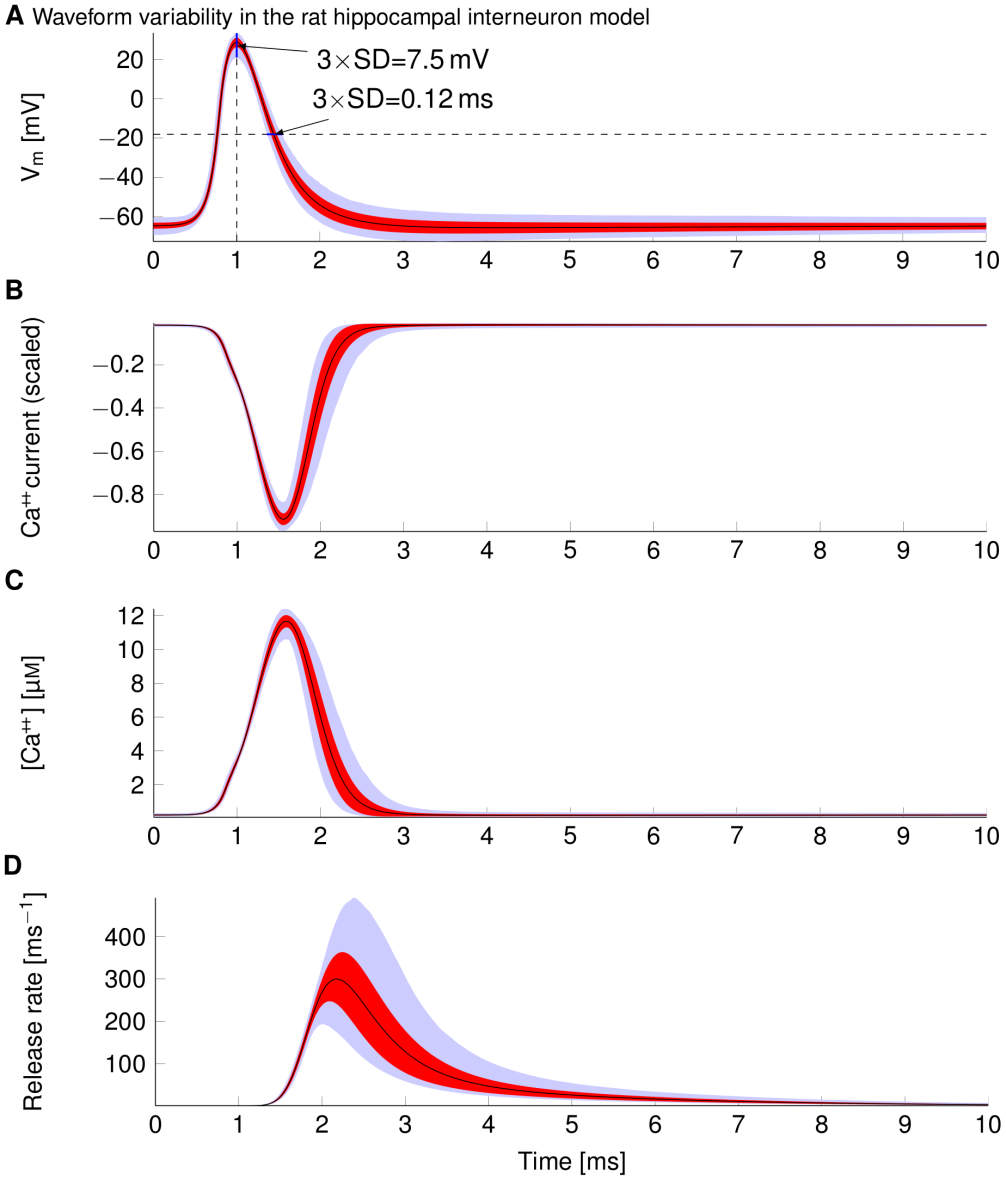
Variability in synaptic processes due to fluctuation in AP waveforms. For all subfigures, the mean waveform is plotted in black, SD in red, and 1%–99% quantiles in light blue. (A) Waveforms of 2000 consecutive APs arriving at the terminal end of an axon of 0.2 µm diameter rat hippocampal interneuron model axon. (B) Ca^++^ current resulting from the integration of the above AP waveforms into a model of a pre-synaptic Ca^++^ channel (see text for details). The current is scaled because we are only interested in its waveform. (C) Intracellular Ca^++^ concentration for a large CNS synapse obtained by scaling the Ca^++^ current waveform, and lengthening it. (D) Time course of vesicle release rates computed for a model of a Calyx-of-Held type synapse (see text for details).

Using a naturalistic white noise current stimulus protocol [32] (1 kHz cut-off frequency, see methods), we elicited spike trains for a period of 10 minutes in a 0.2 µm diameter axon (average cerebellar parallel fibre diameter) using the rat hippocampal interneuron model. Note that interspike intervals and AP triggering currents therefore varied as successive APs were triggered (f = 40.8 Hz±42.8 Hz, mean ± SD). At the axon's distal end (measured at approx. 95% of the axon's total length to exclude boundary effects) waveforms showed considerable variation in the AP shape (Figure 7.A).

Plotting pairs of an AP's width measured at the mid and distal position revealed an uncorrelated structure (correlation coefficients 0.04 and 0.03 for AP width and height), as in the case of single APs. AP widths measured at half-peak had a coefficient of variation (CV = SD/average) of 6% (0.7 ms±0.04 ms) and AP amplitude (resting potential to peak) had a CV of 3% (93.7 mV±2.5 mV). AP waveform variability in spike trains was larger than in the case of individual spikes propagating. The standard deviation of change in AP height after propagating for 1 mm in the axon was 3.5 mV (0.05 ms for the width, N = 2000), compared to 1.6 mV (0.04 ms for the width, N = 250) for the single spike protocol. The profile of waveform variability (Figure 7.A) peaks close to AP threshold and, at a higher level, in the late repolarising phase. Random waveform variability has matching profiles in the spike train and the single AP protocol as it is caused in both cases by the AP mechanism itself.

This illustrates that AP waveform variability is a constantly acting random process, occurring independent of AP initiation or stimulus. Stochastic waveform variability is non-existent in identical simulations where we replace stochastic ion channel models by the equivalent deterministic Hodgkin-Huxley type conductance models. Thus, all axonal variability observed here must result from the effects of the only source of noise modelled – channel noise in Na^+^ and K^+^ channels. We have previously shown that the memory of voltage-gated ion channels causes an increased effect on membrane potential noise, affecting the speed of propagation [18]. The same mechanism is also acting on the waveform.

We have thus quantified the impact of axonal channel noise on AP waveform variability and explained the biophysical mechanisms acting in thin axons. This previously overlooked effect will only become relevant at the neural circuit and behavioural level if it can influence synaptic transmission. Therefore, we modelled next the synaptic transmission process from arrival of the AP to the post-synaptic response.

### Variability in synaptic transmission

Synaptic transmission follows a general sequence of events leading to a post-synaptic response. An AP propagates down the axon and causes the opening of voltage-gated Ca^++^ channels resulting in the influx of Ca^++^ at the pre-synaptic terminal. Ca^++^-sensitive proteins trigger the fusion of vesicles, which release neurotransmitters into the synaptic cleft. These transmitters diffuse and trigger the opening of ion channels in the post-synaptic cell, producing a voltage response. Thus, AP waveform variability could perturb post-synaptic responses [33].

We estimate the synaptic impact of waveform variability for spike trains propagating down a 0.2 µm diameter axon using two distinct approaches: first, we model the individual stages of synaptic transmission in a synapse driven by our thin axons using biophysical models. Second, we use experimental data relating AP width and height to post-synaptic response amplitude to estimate directly how the variability of the AP would transform into response variability.

#### Synaptic variability from signal transduction model

There are few quantitative models of the pre-synaptic mechanisms that link AP arrival to vesicle release. We use one of the most biophysically and quantitatively detailed available models, that of the Calyx-of-Held synapse (reviewed in [34]). The Calyx-of-Held is a very large synapse driven by a reliable, thick axon terminal (1.5–2 µm average diameter [35]). Thus, care has to be taken when extrapolating results from this synapse to small synapses innervated by thin axons. However, due to the rapid Ca^++^ dynamics, the Ca^++^ concentration closely follows the Ca^++^ current and the impact of synapse size is therefore small (see Discussion for details).

**Figure 8:**
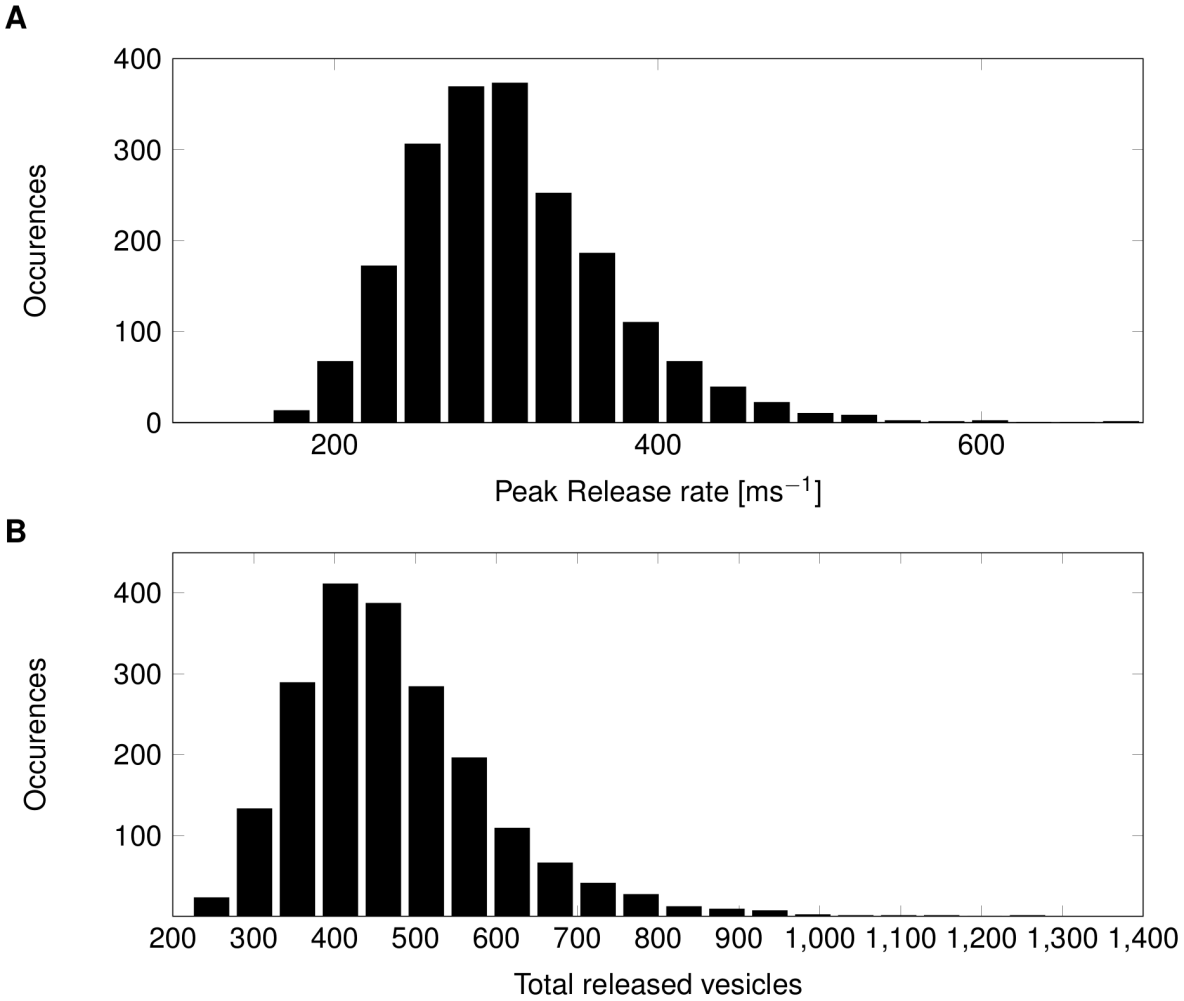
Calculated distribution of synaptic response variability. (A) Distribution of peak vesicle release rate in a large CNS synapse resulting from variability in (N = 2000) AP waveforms (see text for details). (B) Distribution of the total number of released vesicles in a model of Calyx-of-Held type synapse for (N = 2000) AP waveforms.

Our simulations show that both Ca^++^ peak current and total Ca^++^ influx are subject to considerable variability with a CV of 3% and 9% respectively (Figure 7.B). The total inflow of Ca^++^ into the synapse and the associated increase in intracellular Ca^++^ concentration are subject to significant variability (Figure 7.C). Waveform variability in the repolarizing AP phase - varying AP width - leads to a considerable spread in the total influx of Ca^++^. This translates into considerable variability in vesicle release rate and the total duration of release (Figure 7.D), which mainly results from the second, broad variability peak of the (repolarising) AP waveform (see above). The variability in the release time course is due to variations in the AP width, and not to variability in release probabilities [36]. Integrating the instant vesicle release rates of our model yields the total amount of vesicles released for a given AP waveform (Figure 8.B). The variability of the total number of released vesicles is considerable as the CV is about 26% (mean 471 and SD 121). Excitatory post-synaptic current (EPSC) variability can be directly estimated from vesicle release because experimental data has shown that under conditions of normal release probability, released quanta linearly summed to EPSCs [37], [38]. This would suggest an EPSC variability of approx. 25% based on the Calyx-of-Held model if this synapse was innervated by an axon as thin as cerebellar parallel fibres (0.2 µm diameter). We summarise the impact of AP waveform noise on successive stages of synaptic transmission for the Calyx-type synapse model in Table 1.

**Table 1:** Computed signal variability in the successive stages of synaptic transmission for a Calyx-of-Held type synapse (Coefficient of Variation, CV = SD/MEAN) driven by a 0.2 axon.

Stage	Signal	CV
Axon	AP width	6%
Pre-synaptic	Ca^++^ peak current	3%
Pre-synaptic	Ca^++^ influx	9%
Synapse	Peak [Ca^++^]	3%
Synapse	Vesicle release rate	10%
Synapse	Vesicles released	26%
Post-synaptic	EPSC amplitude	≈25%

#### Synaptic variability using direct estimation

To obtain a second, independent estimate of the impact of axonal waveform variability on synaptic variability we looked at experimental data from much smaller synapses. We used published patch-clamp data that directly links AP width to EPSC amplitude in Granule-to-Purkinje Cell synapses. Sabatini et al. [11] report a pronounced power-law relationship between AP width and EPSC amplitude for this synapse. We can use this relationship to translate waveform variability directly into synaptic response variability by combining our results on AP width variability and Sabatini et al.'s Figure 11. Our thin axon APs showed a CV of 6% for AP width, which translates for the Granule-Purkinje Cell synapse to a CV of approx. 25% for the expected EPSC amplitude. Similarly, the computed total Ca^++^ current CV would translate into an EPSC amplitude CV of approx. 25%.

Thus, the predicted synaptic variability from axonal noise from both our synaptic signal transduction model of the (much larger) Calyx-type synapse and the much smaller cerebellar synapse would translate AP waveform fluctuation in thin axons into a CV of 25–30% for their post-synaptic responses. Note, this amount of variability results from axonal noise alone, as we have not accounted for stochastic processes inside the synapse, such as stochastic Ca^++^ channels, the vesicle release signalling cascade, neurotransmitter diffusion or post-synaptic receptors.

## Discussion

Axons are often thought of as fast, reliable transmission channels for electrical impulses. This is mainly because our understanding of axons and action potentials comes from studies of large axons, where noise sources have little impact. However in the many thin (<1 µm diameter) axons in our body, e.g. the dense wiring of cortex or the hundreds of kilometres of C-fibres in our PNS, axonal noise will have significant impact as dictated by basic biophysics [15(review)], [18],[20]. This theoretical work and recent advances in experimental methods have prompted us to reconsider these assumptions [27], [39], [40], and linked to the potential role of the axon as an information processing unit in its own right (reviewed in [41], [42]). Axonal information processing is closely related to the question of how APs are translated at the synapse. Do synapses consider incoming APs as unitary events, or do they use information contained in the waveform (regardless of its origin) to modulate the release of neurotransmitters? Can different synapses sitting on the same axon transmit information differentially? Does the size of axons influence the variability of downstream post synaptic potentials (PSPs)? Understanding these questions has important consequences for the computational capacity of neural circuits, the rate of information transmission between neurons and thus the metabolic efficiency of neurons [43]. We show here that the answer to this question is likely to depend on the diameter, i.e. the anatomy, of axons and is crucial to understand the design constraints of densely wired neural circuits.

### Action potential waveform variability in thin axons

The AP that drives the synapse has to travel along an axon, yet the impact of axonal noise sources on the AP waveform in thin axons was, complete propagation failures set aside [44], not considered in previous studies. Thus, synaptic response reliability and variability [45]–[48] have been in general attributed to mechanisms inside the synapse alone [44]. The results presented here show that in thin unmyelinated axons below 1 µm diameter, commonly found in the CNS and PNS, the travelling waveform of an AP undergoes considerable random variability. This random variability is caused by axonal Na^+^ and K^+^ channel noise, which continuously acts during propagation and thus accumulates with distance [18]. The variability of AP width and amplitude, key parameters linked to synaptic efficacy, dramatically increased (the CV increasing by a factor of approx. 4, see Figure 5) as diameter decreased from 1 µm to 0.2 µm. We predict this change by deriving a scaling relationship which is the direct result of the geometry and general biophysics of axons and thus independent of specific channel kinetics or other biophysical parameters [20]. Invariably, channel noise is bound to increase as diameter decreases to the point that it affects the waveform of the AP. Therefore, we can observe the effects of this variability in CNS and PNS axons, in both vertebrates and invertebrates.

The range of the waveform fluctuations is about 4 to 6 times the SD, thus we found that AP widths vary by 0.1–1 ms in axons between 0.2 and 1 µm diameter. AP width fluctuations result mainly from K^+^ channel noise and inactivating Na^+^ channels during the repolarising phase of the AP. While Na^+^ channel noise principally effects AP propagation speed and thus spike timing reliability [18], K^+^ channel noise has more impact on waveform variability (Although variability in Na^+^ and K^+^ channels partially compensate each other [20]). This fits well with genetic knock-out studies where one type of K^+^ channels was removed from the central nervous system, and which showed increased temporal response jitter [49].

Activity-dependent modulation mechanisms specific to the pre-synaptic terminal are well-known and provide neurons with means for positive or negative feedback regulation of pre-synaptic Ca^++^ influx through regulation of the AP width at the synapse [50]–[53]. One example of such modulatory mechanism, the broadening of APs during spike trains due to slow deactivation of A-type K^+^ channels in mossy fibres has been observed at the level of the synapse [54], and postulated in the axon [41]. This mechanism can be disrupted by random opening of Na^+^ channels in the repolarising phase (which broadens the AP) or random opening of K^+^ channels (which shortens the AP), independently of the spiking history.

### Synaptic variability from noisy action potentials

In general, the observable variability in synaptic responses could be due to two sources: (1) noise and/or (2) very complex mechanisms that appear random. We can distinguish to which extent these two sources of variability are present at the cellular level, by aggregating the effect of random variability generated by identified molecular stochastic processes (such as thermodynamic fluctuations in molecular conformations, reviewed in [15]).

Here, we considered axonal noise as a source of synaptic variability due to channel noise in axons. We modelled a Calyx-of-Held synapse and used data on the Cerebellar Granule-to-Purkinje synapse to estimate the effects of AP waveform noise on synaptic responses in the absence of detailed models for small synapses. Quantitative measurements and models of the mechanistic level of synaptic transmission are limited in small synapses by the technical difficulties to record from thin axon terminals (<1 µm diameter [27]) and the need to look at very short range connections (<500 µm). Therefore, we ignored pre- and post-synaptic activity dependent effects – which may reduce the effects of waveform variability – and used simplified synaptic transmission models.

Care has to be taken when extrapolating results from these synapses to small CNS synapses [55], [56], and extrapolating from any type of synapse to another – even synapses from the same parent axon – may be difficult when details are considered [57]. Bearing that in mind, individual active zones in the Calyx are known to be ultra-structurally similar to those found in small, bouton-like CNS synapses [58]–[60] and the Calyx's functional organization corresponds to a parallel arrangement of several hundred conventional active zones in a single – bouton-like – terminal [61].

Mapping our AP waveform variability data for parallel-fibre like axons onto the empirical relationship between AP width and EPSC amplitude [11] showed a CV of approx. 30% for EPSC amplitude. Other synapses also display this common power-law relationship between AP width and synaptic response, suggesting that axonally induced random variability of the waveform would scale accordingly [5], [6], [11]. The detailed allosteric model of vesicle release rate for Calyx-type synaptic transmission produced comparable amplification of the AP waveform noise (CV increased from 6% to 25%).

Empirical synaptic response CV is typically between 20 and 60%. In all cases modelled here the extrapolated post-synaptic variability is considerable (CV 10 to 30%) and suggests that the observed synaptic variability could be partially explained by axonal noise. Axonal variability will show more impact in synapses placed 1 mm and more down the axon; yet, due to the technical difficulties of finding cell pairs at these distances, their variability is little studied.

The Hodgkin-Huxley axon model and related deterministic axon models allow information about the stimulus to be retained in the AP waveform, e.g. stimulus strength is correlated with AP height [62]. It has been shown **in vitro** that APs triggered and measured at the soma of the same cell can indeed encode information about the stimulus [63], [64]. Changes in the width of APs, whether due to a depolarized soma [65] or application of glutamate [27], have been shown to influence post-synaptic potentials (PSPs). Moradmand et al. [31] studied the deterministic transformation of propagating AP waveforms in a paired-pulse framework and showed that the second AP waveform in the pair becomes increasingly stereotyped due to refractory interaction with the first AP. Thus, even in bursts, only the first spike would be the likely candidate to carry stimulus information in the waveform over long distances. Kole et al. [40] have shown that changes in the waveform of APs operated at the AIS are conserved along the axonal arbour for relatively large diameter axons. Here, we show that for both single APs and spike trains, channel noise decorrelates the waveform (within the limits of the AP's regenerative dynamics) as the AP propagates even over short distances of less than 0.5 mm. Thus, any **en-passant** synapses along the path of an AP will be driven by randomly differing waveforms and produce different responses (even if the synapses were identical [66]).

Synapses innervated by thin axons may have developed mechanisms to circumvent the problem of axonal variability. A simple solution would be to treat an incoming noisy AP waveform as a unitary event. Taking this view, small synapses on thin axons should treat APs as unitary signals and adjust their transduction mechanisms accordingly to be robust to axonal noise effects. This is, in addition to spontaneous APs, another way in which noise constraints affect neural coding [67].

Both axonal and synaptic noise will affect post-synaptic response amplitude variability and vesicle release probability. The above results suggest that the amount of random variability depends on axonal and synaptic morphology. Impedance matching and volume conservation in densely packed CNS tissues suggest that the diameter of axons should be closely related to the size of the synapse (If the synapse was much larger, the impedence mismatch would cause a drop in the membrane potential, preventing incoming APs from triggering vesicle release.) Although there exists little data relating axon length and diameter to synaptic morphology and function, one can see that the input resistance of synapses is proportional to their surface area. Similarly, the voltage-sensitivity to an incoming AP increases with the inverse of the squared synaptic diameter. Thus, a smaller synaptic geometry supra-linearly amplifies variations in Ca^++^ current and influx, and due to the smaller volume, variations in Ca^++^ concentration as well. Thus, if the properties and kinetics of signal transduction were invariant to synaptic size, an increase in synaptic response variability due to noise in pre-synaptic signal transduction would be expected for smaller synapses. Variability in the waveform of action potentials is known to also affect synaptic latency [68]. However, a careful investigation of this phenomenon requires stochastic simulations of both Ca^++^ channels and the 5-state vesicle release model.

Axons play an important active role for information processing that may be comparable to that of dendritic computation [41]. However, axonal variability has traditionally not been considered as a source of neuronal variability [69] because the AP mechanism was considered highly reliable by extrapolating from classic studies in large (3 orders of magnitude larger diameters) fibres such as squid giant axons [70]. Yet, in densely connected central neural circuits the APs become sensitive to channel noise [18]. The effects of channel noise will inescapably increase non-linearly as diameter decreases due to the very nature of the AP mechanism [20]. Axonal channel noise will affect the reliability (<0.1 µm diameter) and cause considerable variability to both timing (<0.5 µm diameter) [18] and, as shown here, the shape of the AP in axons below 1 µm diameter. Thin unmyelinated axons typically innervate large numbers of small CNS synapses [71] and are associated with, and required for, the high level and density of circuit miniaturisation encountered in the cortex and the cerebellum [20], [72].

In sensory and motor nervous systems, reliability is typically achieved by averaging over many release sites and high release rates. The corresponding large synapses are associated with large axons. However, in the cerebral cortex, hippocampus and cerebellum the dense connectivity within a restricted space limits the diameter of axons, the number of redundant axonal connections and the size of the synaptic contact areas. This makes synaptic transmission prone to the effects of axonal channel noise in thin axons innervating small synapses. The results presented here prompt careful experimental consideration, because paired-cell measurements and optical methods do not offer the control and resolution necessary to determine the source of PSP variability. More generally, we show how molecular noise sources can explain observed variability at higher levels of biological organisation.

## Methods

Simulations were based on biophysical data and reproduced physiological data such as the amplitude and width of APs (but see [73], for possible shortcomings and other models). Computations were carried out using the Modigliani stochastic simulator [74], available from http://www.modigliani.co.uk, on a Linux PC using an Intel core i7 processor with the binomial algorithm [19]. Simulations were carried out using Markov models of squid giant axon channels (Na^+^ channel expressed by gene GFLN1, K^+^ channel expressed by gene SqKv1.1) as several independently constructed kinetic models exist for these channels. We used the original models given by Hodgkin 18 and Huxley [75] as well as a more recent model with delayed opening [76] with little difference in results. These ion channel models captured the corresponding ion channel kinetics from patch-clamp experiments. To account for differences between the squid giant axon and mammalian axon [77], [78], we confirmed the results using models of rat hippocampal interneurons with Markov models of rodent ion channels [79] shown to have little overlap between Na^+^ and K^+^ currents [78], and a model of rodent C-fibre axons (Nav1.8) [80]. We chose to simulate all model axons at the temperature at which their channel kinetics were experimentally recorded. The parameters for each model are summarised in Table 2.

**Table 2:** Simulation parameters. SGA stands for squid giant axon. RHI stands for rat hippocampal interneuron.

Parameters	SGA-HH	SGA-Patlak	RHI	C-fibre
Membrane capacitance [µF cm^−2^]	1	1	1	0.81
Axial resistance [Ω cm]	35.4	35.4	70	70
Leak conductance [mS cm^−2^]	0.3	0.3	0.1	0.14
Na^+^ single channel conductance [pS]	20	20	15	20
Na^+^ channel density per µm^2^	60	60	23	62.5
K^+^ single channel conductance [pS]	20	20	14	17
K^+^ channel density per µm^2^	18	18	6	10
Leak rev. pot. [mV]	−54.4	−54.4	−65	−61.14
Na^+^ rev. pot. [mV]	50	50	55	79.6
K^+^ rev. pot. [mV]	−77	−77	−90	−85
Temperature [C]	6.3	6.3	35	24

### Stimulus protocols

We used two sets of simulation protocols. In the first protocol, 0.1 µm, 0.2 µm, 0.3 µm, 0.5 µm diameter (1 cm long) and 1 µm diameter (2 cm long) axons were stimulated in a single spike per trial framework (N = 250 trials per diameter) to allow for fast parameter exploration. In the second protocol, we simulated 10 minutes long spike trains. To this end, a 0.2 µm diameter (2 mm long) axon was stimulated with a zero-mean white noise current (SD = 0.01 nA, 1 kHz corner frequency) injected at the proximal end. Membrane properties were set to R_a_ = 70 Ωcm, R_m_ = 20000 Ωcm^2^, typical for cortical cells [81]. All axons had a resting potential of −65 mV. After visual inspection of the data, we used a threshold discriminator detecting AP height and aligned their waveforms at the rising half-peak potential crossing time. We measured voltage-traces of the AP waveforms at regular intervals (typical distance 10% of total axon length, see Figure 1.A) between 5% and 95% of the axon's length (0% being the axon's proximal end) to avoid measuring stimulus artefacts or boundary effects and to measure the evolution of the AP shape along the axon. The height and width of APs were defined according to Figure 1.C.

### Modelling synaptic transmission

We estimated the impact of action potential waveform variability on synaptic transmission using two approaches. First, synaptic transmission was modelled using data and deterministic models from the Calyx of Held synapse (reviewed in [34]). We drive the Calyx- of-Held synapse with noisy spike train waveforms directly (voltage clamping the synapse) to circumvent any potential issues of impedance mismatch. We then compute Ca^++^ currents evoked by the AP waveform by integrating the dynamics of a Hodgkin-Huxley type conductance-based Ca^++^ channel model of Calyx synapses [82]. We describe the Ca^++^ channel behaviour using a conductance-based Hodgkin-Huxley type model with two identical gating particles (denoted m) with channel opening probability 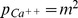. The corresponding rate functions are 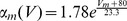 and 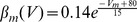, with dynamics 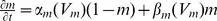 [83]. Here we modelled the voltage-gated Ca^++^ channel deterministically, and calculated the waveform of the incoming Ca^++^ current using a reversal potential of 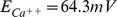. This Ca^++^ current model simplifies the heterogeneity in both the biophysical and the pharmacological properties of Ca^++^ currents found in Calyx-type synapses [84], [85], but was shown to capture sufficient detail for quantitative modelling of synaptic transmission [82].

**Figure 9:**
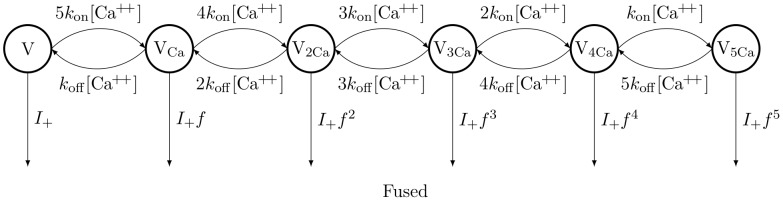
Allosteric model for the instantaneous rate of vesicle release in a Calyx of Held. This model is used to estimate the vesicle release rate as a function of local transient Ca^++^ concentration. Transition rates between states depend on Ca^++^ local concentration in the vicinity of the vesicles. The release probability is higher the more Ca^++^ ions are bound to the vesicle, according to a factor *f* = 31.3. The base rate constant *I_+_* is set to 2×10^−4^ s^−1^.

The transient 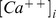 encountered by vesicles in the proximity of Ca^++^ channels is shown to follow a time course similar to that of the Ca^++^ current [86], [87], i.e. the Ca^++^ concentration rapidly declines due to effects such as buffering [88]. We use the dynamics of Ca^++^ channels and modify them slightly so that the rise time is conserved, but the width at half-height becomes approx. 100 µs longer [86]. We then scaled the resulting waveform to have a peak of approx. 12 µm. This value was chosen based on an approximate reading of figure 6.B in [89]. The resting 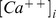 was 50 nm. Finally, we modelled the impact of 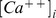 on transmitter release using an allosteric “5 state” model [90], which allows us to derive the instantaneous vesicle release rate (Figure 9).

Synaptic transmission dependence on the AP waveform was also modelled using experimental data for the much smaller rodent cerebellar Granule cell-to-Purkinje cell synapse. In this synapse the width of the pre-synaptic AP waveform, pre-synaptic Ca^++^ entry and the resulting post-synaptic currents were directly measured [11]. To obtain an estimate of how AP waveform variability would affect this synapse, we passed the simulated APs' widths through the experimentally characterised relationship between postsynaptic response and AP width. We then computed the variability of the post-synaptic response over all APs.

## Supporting Information

Figure S1 Correlation coefficient of waveform variability as a function of distance. Correlation coefficient of the difference between individual AP waveforms and the mean AP waveform recorded as a function of distance between record locations for 0.2, 0.5 and 1 micron diameter axons.
(TIF)

Figure S2 Peak of variability in waveforms of APs aligned at 20% of the AP peak. The peak of AP waveform variability is in the same position than in Figure 4, where APs were aligned at 50% of AP peak. This figure is produced in the same fashion as subpanels A and B from Figure 4, but AP waveforms have been aligned at 20% of the AP amplitude. (A) Typical shape of an action potential in the squid giant axon. (B) The variability in the waveform at each moment in time (N = 250). We define the variability as 3×SD of the membrane potential at each point in time.
(TIF)

Material S1 Modigliani configuration files for simulating the squid giant axon. All data required for other axons can be found in the methods sections, and related papers cited in that section.
(ZIP)
