# Supplementary figures and images for: Axonal Noise as a Source of Synaptic Variability
Source: PLoS Comput Biol. 2014 May 8;10(5):e1003615. doi: 10.1371/journal.pcbi.1003615 (PMC4014398; doi:10.1371/journal.pcbi.1003615)

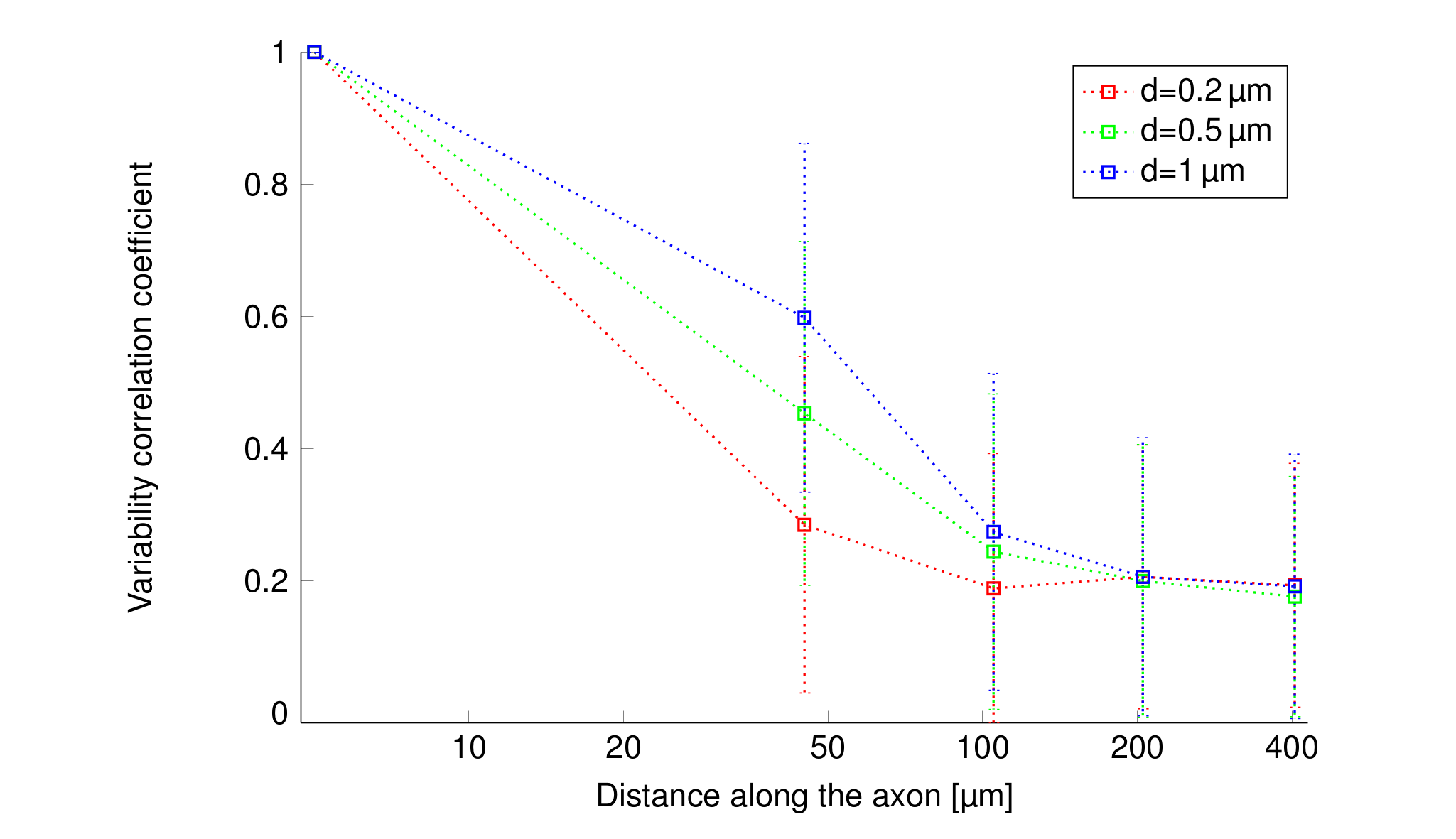

Supplement: Figure S1 — Correlation coefficient of waveform variability as a function of distance. Correlation coefficient of the difference between individual AP waveforms and the mean AP waveform recorded as a function of distance between record locations for 0.2, 0.5 and 1 micron diameter axons. (TIF) [file pcbi.1003615.s001.tif]

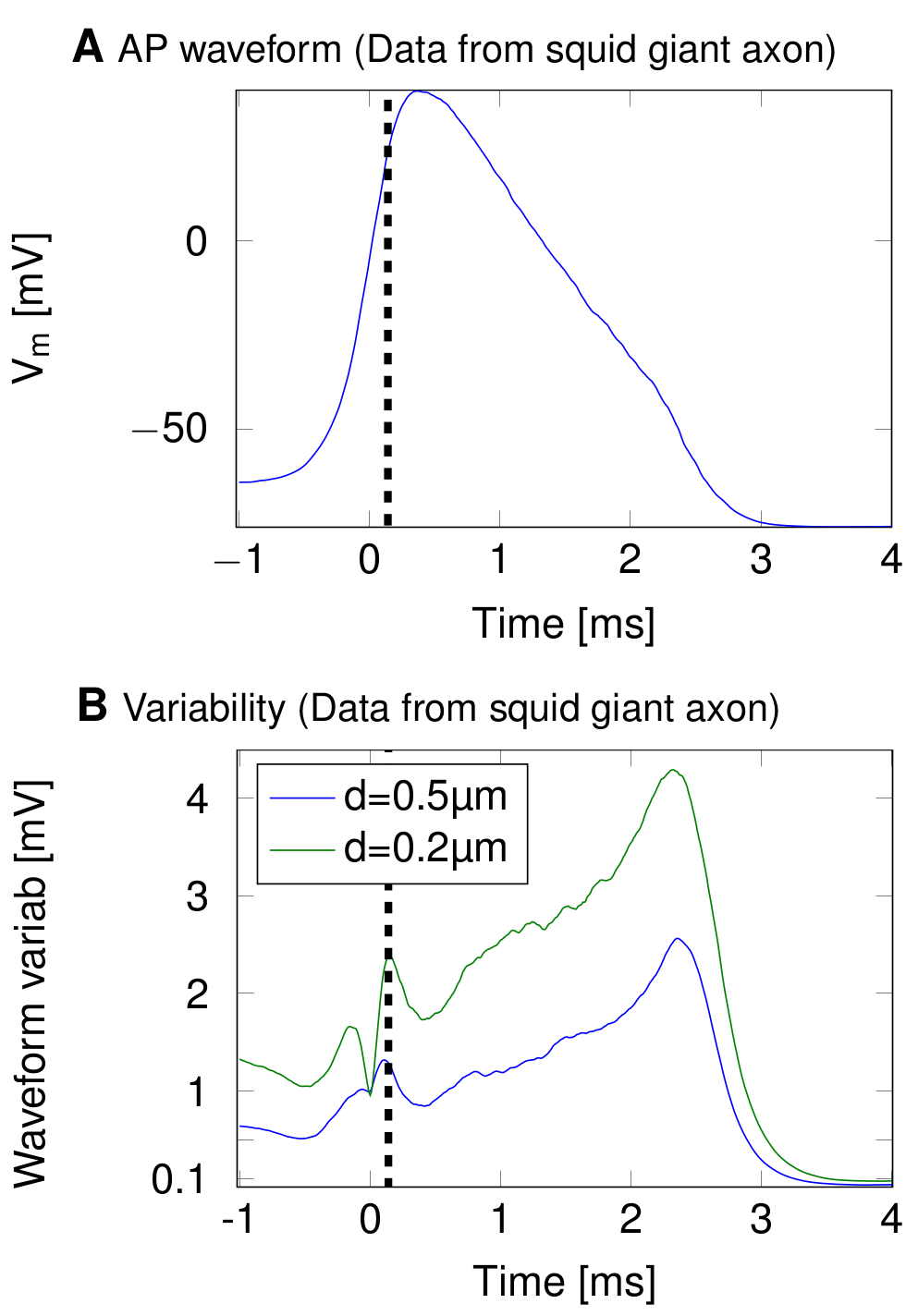

Supplement: Figure S2 — Peak of variability in waveforms of APs aligned at 20% of the AP peak. The peak of AP waveform variability is in the same position than in Figure 4, where APs were aligned at 50% of AP peak. This figure is produced in the same fashion as subpanels A and B from Figure 4, but AP waveforms have been aligned at 20% of the AP amplitude. (A) Typical shape of an action potential in the squid giant axon. (B) The variability in the waveform at each moment in time (N = 250). We define the variability as 3×SD of the membrane potential at each point in time. (TIF) [file pcbi.1003615.s002.tif]
